# Correlation Between High-sensitivity C-reactive Protein and Reactive Oxygen Metabolites During A One-year Period Among Asymptomatic Subjects

**DOI:** 10.4021/jocmr755w

**Published:** 2012-01-17

**Authors:** Kazuhiko Kotani, Nobuyuki Taniguchi

**Affiliations:** a Department of Clinical Laboratory Medicine, Jichi Medical University, Shimotsuke-City, Tochigi, Japan

## Abstract

**Background:**

Inflammation and oxidative stress are associated with human health and the disease status. The present study aimed to investigate the longitudinal correlation between the diacron reactive oxygen metabolites (d-ROMs) level, as an oxidative stress-related marker, and high-sensitivity C-reactive protein (hsCRP), as an inflammatory marker, during a one-year period among asymptomatic subjects.

**Methods:**

The data, including anthropometric and biochemical markers, were collected at baseline and after the one-year period from 71 participants (male/female = 41/30, mean age 50 years). The correlation between the changes of the d-ROMs and hsCRP levels during the study period was examined.

**Results:**

A simple correlation analysis showed a significant and positive correlation to exist between the changes of the d-ROMs and hsCRP levels (r = 0.40, P < 0.01). This significant correlation remained independent in a multiple linear regression analysis adjusted for confounding factors.

**Conclusions:**

The present findings suggest that the relationship between the d-ROMs and hsCRP levels could be prospectively followed, and that monitoring both markers may help to better understand the cooperation of inflammation and oxidative stress in association with health and disease. Further studies are necessary to clarify the biological mechanism(s) responsible for the observed relationship.

**Keywords:**

Oxidative stress; Oxygen reactive species; Inflammation; CRP

## Introduction

In recent years, chronic inflammation, as assessed by the C-reactive protein (CRP) level, and oxidative stress, as assessed by oxidative stress-related markers, have attracted much attention as non-traditional pathological factors, since these conditions have been shown to play important roles in human health and disease [1-5]. While the measurement of high-sensitivity CRP (hsCRP) in blood is currently commonplace for evaluating inflammation, the oxidative stress-related markers that can be used to easily evaluate the oxidative stress status in the clinical settings remain under consideration [6].

The diacron reactive oxygen metabolites (d-ROMs) test was recently introduced, and this measurement can quantify the oxidative stress status by measuring the levels of hydroperoxides of global organic compounds (lipids, proteins, nucleic acids, etc.) [7,8]. This test is currently used as an easy clinical marker for evaluating oxidative stress. In fact, a few studies have reported a significantly positive relationship between the d-ROMs and hsCRP levels in patients at high risk for coronary artery disease [9] and in general populations [10-12]. However, these studies were based on a cross-sectional design; therefore, prospective studies are required to confirm their relationships. The aim of the present study was thus to investigate the longitudinal correlation between the d-ROMs and hsCRP levels in an asymptomatic population.

## Methods

This one-year observational design study consisted of 71 asymptomatic subjects (male/female = 41/30, mean 50 ± 7 years) who had health check-up examinations both at baseline and after the one-year period. The inclusion criteria were non-smokers and participants not taking any medications or supplements. The exclusion criteria were participants who had acute infectious diseases, such as a common cold, or who had a history of cardio/cerebrovascular, thyroid, collagen, severe kidney or liver diseases. This study was approved by the Institutional Ethics Committee, and all subjects gave their informed consent.

At baseline and after the one-year period, we obtained data from each subject in an overnight fasted state. The body mass index (BMI) was calculated based on the weight and height measured while subjects wore light clothing without shoes. The systolic blood pressure (SBP) and diastolic blood pressure (DBP) were measured in the subject’s right arm with a mercury sphygmomanometer while the subject was the seated position, and then, the mean blood pressure (MBP) was calculated by the equation of DBP + [(SBP-DBP)/3]. The serum total cholesterol (TC), triglyceride (TG), HDL-C and fasting plasma glucose (FPG) levels were measured by standard enzymatic methods. The serum hsCRP levels were measured by an enzyme-linked immunosorbent assay (Assaypro Co. Ltd., St. Charles, MO, USA). The d-ROMs levels were measured using a kinetic spectrophotometric assay (the F.R.E.E system; Diacron Srl., Grosseto, Italy) with intra- and inter-assay coefficients of variation of 2.1% and 3.1%, respectively [7,8]. Briefly, serum samples were mixed with a buffered solution, and a chromogenic substrate was added to the mixture. The mixture was centrifuged, and then incubated in a thermostatic block. The absorbance was recorded at 505 nm. The measurements are expressed in U. Carr., where 1 U. Carr. corresponds to 0.08 mg/dL H_2_O_2_.

The data are expressed as the means ± standard deviation (SD) or the medians plus the interquartile range. The data between at baseline and after the one-year period were compared using paired t-tests. Changes in the respective measurements during the one-year period were calculated by subtracting the values at baseline from the values after the one-year period. A simple correlation test (Pearson’s test) and a multiple linear regression analysis, adjusted for all the other factors, were used to observe the correlation between the changes of the d-ROMs and hsCRP levels during the one-year period. The TG and hsCRP values were log-transformed in these analyses because of their skewed distributions. Statistical significance was defined as a P-value < 0.05.

## Results

After the one-year period, the hsCRP levels showed a small but significant increase [0.026 (0.013 - 0.049) to 0.031 (0.017 - 0.068) mg/dL; P = 0.03], while the d-ROMs levels showed a non-significant and slight increase (311 ± 45 to 317 ± 47 U. Carr.; P = 0.15). The mean/median levels of the other measured factors between the values at baseline and post-one-year period were largely unchanged: with changes from 22.5 ± 3.0 to 22.6 ± 3.1 kg/m^2^ in the BMI (P = 0.29), 93 ± 13 to 93 ± 11 mmHg in MBP (P = 0.52), 5.32 ± 1.33 to 5.25 ± 0.91 mmol/L in TC (P = 0.51), 1.61 ± 0.41 to 1.62 ± 0.43 mmol/L in HDL-C (P = 0.42), 0.92 (0.71-1.39) to 1.02 (0.67-1.49) mmol/L in TG (P = 0.59), 5.38 ± 0.54 to 5.52 ± 0.77 mmol/L in FPG (P = 0.06).

A simple correlation analysis showed that there was a significant and positive correlation between the changes of the d-ROMs and hsCRP levels (Table 1). The changes of the d-ROMs or hs-CRP levels were significantly correlated with those of the BMI (positively) or of HDL-C (inversely). A subsequent multiple linear regression analysis revealed that there remained a significant and positive correlation between the changes of the d-ROMs and hsCRP levels, independent of the changes of the other measured factors.

**Table 1:** The Correlation Between the Changes of the D-ROMs and HsCRP Levels During A One-year Period

	**D-ROMs (dependent factor)**		**HsCRP (dependent factor)**	
	**r (P)**	**β (P)**	**r (P)**	**β (P)**
Age, years	0.13 (0.30)	0.13 (0.31)	0.18 (0.14)	0.18 (0.15)
Gender, male	0.01 (0.98)	-0.01 (0.93)	0.05 (0.69)	0.03 (0.83)
Body mass index, kg/m^2^	0.25 (0.04)*	0.11 (0.42)	0.33 (0.01)*	0.24 (0.06)
Mean blood pressure, mmHg	0.23 (0.06)	0.20 (0.12)	0.13 (0.29)	0.04 (0.73)
Total cholesterol, mmoL/L	-0.12 (0.31)	-0.09 (0.47)	-0.11 (0.35)	-0.06 (0.67)
HDL cholesterol, mmoL/L	-0.25 (0.03)*	-0.13 (0.33)	-0.28 (0.02)*	0.14 (0.30)
Triglyceride, mmoL/L	0.01 (0.96)	-0.09 (0.48)	0.10 (0.43)	0.01 (0.97)
Plasma glucose, mmoL/L	0.13 (0.27)	0.09 (0.43)	0.09 (0.48)	0.02 (0.89)
HsCRP, mg/dL	0.40 (< 0.01)*	0.27 (0.04)*	-	-
D-ROMs, U. Carr.	-	-	0.40 (< 0.01)*	0.26 (0.04)*

D-ROMs: diacron reactive oxygen metabolites; HsCRP: high-sensitivity C-reactive protein; HDL: high-density lipoprotein. r: simple correlation test (Pearson’s test), β: multiple linear regression analysis, adjusted for all the measured factors. The triglycerides and hsCRP values were log-transformed because of their skewed distributions. Significance level: *P < 0.05.

## Discussion

The present study found that there was an independent, significant and positive correlation between the changes of the d-ROMs and hsCRP levels during a one-year period among asymptomatic subjects. Although the cooperation of oxidative stress and inflammation is a recent key concept developed as a result of a deeper understanding of the pathophysiology of health and disease [1-5], the correlation between the d-ROMs and hsCRP levels has so far been examined only in cross-sectional studies [9-12]. From the viewpoint of establishing evidence, it is valuable to note that the present prospective study confirmed the coexistence of oxidative stress and inflammation, even though the longitudinal period of the study was relatively short.

Although the detailed biological mechanism (s) underlying the present results remain unclear, there are several possible explanations. Oxidative stress, which results from an imbalance in the oxidative/antioxidative system (i.e., caused by an unhealthy lifestyle), and inflammation can both induce an oxidative stress response and inflammatory molecules via cell dysfunction in systemic organs, including the adipose tissue, liver and vasculature [1,3]. Furthermore, the pathways form a vicious cycle of oxidative stress and inflammation [13]. The oxidative stress-inflammation inter-relationship is even present in asymptomatic subjects, and this may partially explain the correlation between the d-ROMs and hsCRP levels.

There were some limitations to the present study. The observational period was relatively short, while this can have merit because it can reduce the impact from the fluctuation of various factors influencing inflammation and oxidative stress (e.g., weight cycling). The sample size was small, and the study population was basically restricted to the non-severe diseased participants. Future studies with a long-term prospective design and with larger and more varied populations are needed.

In summary, the present study confirmed that there was an independent, significant and positive correlation between the changes of the d-ROMs and hsCRP levels during a one-year period among asymptomatic subjects. The relationship between the d-ROMs and hsCRP levels was prospectively followed, and monitoring both markers may help to better understand the cooperation of inflammation and oxidative stress in association with health and disease. Further studies are necessary to clarify the biological mechanism (s) underlying the observed relationship.
